# Editorial: Recent advances in iron excess toxicity and its interaction with metals in plants

**DOI:** 10.3389/fpls.2024.1524947

**Published:** 2024-12-03

**Authors:** May Sann Aung, Andriele Wairich, Felipe Klein Ricachenevsky

**Affiliations:** ^1^ Department of Biological Production, Faculty of Bioresource Sciences, Akita Prefectural University, Akita, Japan; ^2^ Center of Biotechnology, Federal University of Rio Grande do Sul, Porto Alegre, RS, Brazil; ^3^ Botany Department, Institute of Biosciences, Federal University of Rio Grande do Sul, Porto Alegre, RS, Brazil

**Keywords:** iron excess, rice, Arabidopsis thaliana, metal stress, transport

## Introduction

Iron (Fe) is an essential mineral element in all living organisms. It is important for many functions in plants, particularly respiration and photosynthesis, where its ability to gain and lose electrons makes it essential in electron transfer reactions. However, excess uptake of Fe can cause cellular toxicity in plants, as Fe can produce reactive oxygen species through Fenton chemistry. Fe toxicity can lead to poor plant growth and yield, especially in acidic and waterlogged soils. Although not all plants grow in such conditions, environmental changes may cause temporary increases in Fe availability, causing excessive Fe uptake and toxicity, which can decrease plant growth and yield. Especially compared to Fe deficiency, mechanisms of how plants deal with high Fe concentrations are poorly understood. In this Research Topic, we focused on papers that highlight how plants deal with high Fe concentrations in their environment and within their tissues.

Two papers focused on rice (*Oryza sativa* L.) plants, which are frequently cultivated in flooded fields, causing Fe^3+^ reduction to Fe^2+^ and excessive Fe uptake. Wairich et al. thoroughly reviewed the knowledge on how rice plants deal with Fe excess. In particular, the authors discussed different defense types that distinct rice genotypes employ to tolerate high Fe concentrations, compared published studies that used transcriptome analyses to propose core genes that are regulated by Fe excess in rice roots and shoots, and summarized the genes and proteins that are established in the literature as clearly involved in Fe excess response and detoxification. The review is an excellent starting point to move our understanding of Fe excess tolerance in rice forward.

In another study, Rajonandraina et al. showed that Fe excess symptoms in rice plants are mitigated by supplying magnesium (Mg). Authors report that, in the field, plants treated with Mg tended to decrease Fe concentration in shoot tissue, while further experiments in hydroponics demonstrated that both Fe concentration and a biomarker for oxidative stress were not changing significantly, suggesting that Mg reduces leaf bronzing—the hallmark phenotype of Fe excess—by inducing tissue-level tolerance. The study also used RNA-Seq to identify possible shoot mechanisms involved in the Mg-based tolerance to Fe excess and found that NAC transcription factors might be involved. The work provides an interesting example of how nutrient balances might be key to induce Fe excess tolerance in plants and presents extra evidence related to the role of Mg counteracting soil-related stresses in rice plants.

Mai et al. dealt with the problem of how Fe homeostasis-related genes are currently classified by Gene Ontology (GO), a tool frequently used to analyze transcriptome and proteome enrichment of terms associated with molecular functions, cellular components, and biological processes. The authors demonstrate that some of the genes associated with Fe-related terms are not well supported by evidence and suggest that proof from the literature can be used to add missing genes and their respective homologous groups to increase the reliability of GO terms. The paper compiles lists of genes involved in Fe homeostasis with direct experimental evidence, genes regulated by well-known transcription factors, and members of gene families or ortholog/paralog groups that are not well characterized, which can be used in custom enrichment analyses, aiming to eliminate the gaps and marked deficiencies in the listed genes previously available. The work is focused on *Arabidopsis thaliana* and provides an excellent example that could be adapted to crops.

In an experimental work on *A. thaliana*, Zhu et al. demonstrated the key roles of ferroxidases AtLPR1 and AtLPR2 in Fe(II) oxidation to Fe(III) in the vascular tissues, particularly in the xylem. Root-to-shoot translocation of Fe by the transpiration stream is known to occur through Fe(III)–citrate complexes, which depends on root symplast-to-apoplast transport of citrate and Fe(II), performed by AtFRD3 (Durrett et al., 2007) and AtFPN1 (Morrissey et al., 2009), respectively. AtLPR1 and AtLPR2 are involved in the next step, the oxidation of Fe(II) into Fe(III), promoting the formation of xylem Fe(III)–citrate complexes and therefore are important to avoid excessive Fe accumulation in the xylem under Fe sufficient condition, as well as for optimizing Fe distribution under varying Fe concentrations.

In summary, our Research Topic provides both reviews and original research papers that help advance our understanding of Fe nutrition in plants and how plants respond and deal with excessive accumulation of Fe.
